# Assessing the Chronic Environmental Risk of Graphene Oxide Using a Multimarker Approach Across Three Trophic Levels of the Aquatic Ecosystem

**DOI:** 10.3390/nano15201553

**Published:** 2025-10-12

**Authors:** Ildikó Fekete-Kertész, Krisztina László, Anna Bulátkó, Benjámin Gyarmati, Zoltán Molnár, Mónika Molnár

**Affiliations:** 1 Environmental Microbiology and Biotechnology Group, Department of Applied Biotechnology and Food Science, Faculty of Chemical Technology and Biotechnology, Budapest University of Technology and Economics, Műegyetem rkp. 3., H-1111 Budapest, Hungary; molnar.monika@vbk.bme.hu; 2 Surface Chemistry Group, Department of Physical Chemistry and Materials Science, Faculty of Chemical Technology and Biotechnology, Budapest University of Technology and Economics, Műegyetem rkp. 3., H-1111 Budapest, Hungary; laszlo.krisztina@vbk.bme.hu (K.L.); bulatko.anna@vbk.bme.hu (A.B.); 3 Soft Matters Group, Department of Physical Chemistry and Materials Science, Faculty of Chemical Technology and Biotechnology, Budapest University of Technology and Economics, Műegyetem rkp. 3., H-1111 Budapest, Hungary; gyarmati.benjamin@vbk.bme.hu; 4 Department of Plant Sciences, Faculty of Agricultural and Food Sciences, Széchenyi István University, Vár Sqr. 2., H-9200 Mosonmagyaróvár, Hungary; molnar.zoltan@sze.hu

**Keywords:** nanoecotoxicology, graphene oxide, chronic toxicity, environmental risk assessment, aquatic ecosystem

## Abstract

With the rapid increase in the synthesis and application of graphene oxide (GO), questions have emerged about its inadvertent entry into aquatic habitats and the ecological consequences associated with such exposure While several studies have addressed the acute effects of GO, knowledge on its chronic impacts across multiple trophic levels remains limited. In this study, we assessed the chronic toxicity of a well-characterized GO product using model organisms representing three trophic levels: the bioluminescent marine bacterium *Aliivibrio fischeri,* unicellular green algae (*Chlamydomonas reinhardtii*, *Chlorella vulgaris*, *Desmodesmus subspicatus*), the cyanobacterium *Synechococcus elongatus*, and the freshwater cladoceran *Daphnia magna*. Endpoints included bioluminescence inhibition in bacteria, growth inhibition in photosynthetic primary producers, and reproduction and refined physiological parameters (heart rate, feeding activity) in *D. magna*. Our results demonstrated clear concentration-dependent chronic effects of GO, with *A. fischeri*, the applied photosynthetic primary producers and *D. magna* exhibiting significant inhibition of bioluminescence, growth, delayed onset of reproduction, and reduced fitness parameters, respectively. Based on the collected data, a comprehensive ecotoxicological risk assessment was carried out, revealing that pristine GO may pose negligible hazard to aquatic ecosystems under environmentally relevant exposure scenarios. The outcomes clearly demonstrate the relevance of incorporating chronic and multi-trophic effects when evaluating the ecological risks of emerging nanomaterials such as GO.

## 1. Introduction

Graphene oxide (GO) is among the most widely utilized nanomaterial derivatives within the graphene family, obtained through the oxidative exfoliation of graphite [1]. Its growing application has driven research into its potential environmental effects on aquatic and terrestrial ecosystems over the past decade [2,3,4]. The toxicity of both pristine GO and its functionalized forms has been extensively investigated in acute standard ecotoxicity studies, particularly in relation to *Daphnia magna*, a filter-feeding zooplankton commonly used in ecotoxicology and a crucial component of aquatic food chains [2,4,5,6,7].

Toxicity tests with *Aliivibrio fischeri* revealed that short exposure to graphene oxide (GO) can adversely affect the bioluminescence of this marine bacterium, indicating sublethal stress. The results showed a concentration-dependent decrease in light emission over extended exposure periods, suggesting that GO interferes with cellular processes essential for luminescence [8,9,10,11]. These findings highlight the potential for chronic effects of GO even at low concentrations, emphasizing the need for long-term ecotoxicological evaluations in risk assessments of graphene-based nanomaterials.

GO has been shown to inhibit the growth and physiological functions of diverse algal species—including green algae such as *Chlorella pyrenoidosa*, *Raphidocelis subcapitata*, and cyanobacteria like *Microcystis aeruginosa*—via mechanisms involving oxidative stress, membrane damage, nutrient depletion, and shading-induced photoinhibition [12,13]. Recent reviews systematically highlight that GO’s toxicity in algal ecotoxicology stems primarily from its physicochemical properties and interactions with algal surfaces, leading to reduced photosynthetic activity and compromised cell integrity across various species [12,14]. Although these reviews emphasize consistent toxic responses across taxa, they also primarily focus on short-term exposures spanning up to 96 h, potentially overlooking the chronic impacts of prolonged GO exposure on algal physiology and aquatic ecosystem health.

However, there is limited knowledge about the chronic, product-specific toxicity of well-characterized GO products, particularly regarding physiological and behavioral effects. A limited number of studies discuss the chronic effects of graphene-family materials on *D. magna*. In a 21-day long exposure study the impact of graphene and its surface-functionalized derivatives (carboxylated, aminated, hydroxylated and thiolated GO) was studied on the reproduction and development of *D. magna* complemented with RNA sequencing at 1 mg/L concentration, revealing that surface functionalization alleviated the toxicity of graphene nanomaterials to *D. magna* growth and reproduction reletaed to several metabolic pathways (oxidative stress, protein and carbohydrate digestion and absorption) [15].

The toxicological consequences of interactions between engineered nanomaterials (ENMs) and contaminants or their derivatives have been mainly overlooked, due to the limited understanding of the effects of graphene nanomaterials. As knowledge of the toxicological properties of graphenic nanomaterials and their derivatives grows, future research may focus on assessing the chronic toxicity of these materials and the byproducts generated during their environmental transformation. neglected mainly In this research, we investigated the chronic toxicity of a well-characterized graphene oxide nanosuspension using multiple ecotoxicity methods, such as the *Aliivibrio fischeri* bioluminescence inhibition assay, alga and cyanobacterium growth inhibition tests, and the *Daphnia magna* reproduction test with additional physiological endpoints: heart rate and feeding activity. was to address critical knowledge gaps in ecotoxicology and environmental risk assessment related to the impacts of graphene-based nanomaterials. To achieve this, the study emphasized the importance of evaluating these materials under environmentally relevant conditions, particularly across diverse trophic levels in aquatic ecosystems—including microorganisms, algae, cyanobacteria, and aquatic invertebrates.

Effective concentration values were established for each test organism, providing a foundation for the development of preliminary predicted no-effect concentration (PNEC) values. These values contribute to the initial risk characterization of the tested graphene oxide (GO) nanoparticles. The underlying hypothesis was that the safety threshold derived from the comprehensive chronic ecotoxicity assessment would demonstrate that the tested GO suspension poses no significant risk to the aquatic environment.

## 2. Materials and Methods

### 2.1. Synthesis and Characterization of Pristine GO Nanoparticles

GO nanoparticles were synthesized from natural graphite (Graphite Týn, Týn nad Vltavou, Czech Republic; average particle size: 63 µm) using the Hummers’ method, achieving a yield of 33%, as previously described by Gyarmati et al. [16]. To achieve a neutral pH, successive centrifugation (Jouan BR4i Multifunction Centrifuge, Thermo Scientific, USA; 9000 min^−1^) and thorough washing steps were performed using a 1 M HCl solution and distilled water. The GO stock suspension had a concentration of approximately 1 *w*/*w*%. The C/O ratio and surface area of the freeze-dried GO monolith were determined to be 2.6 and 20 m^2^/g, respectively, and TG/MS results indicated thermal stability below 200 °C. Hummers’ exfoliation significantly damaged the integrity of the aromatic graphene layers. The intensity ratio of the characteristic G (graphitic) and D (defect) peaks (IG/ID) in the Raman spectrum decreased from 6.0 ± 0.5 in pristine graphite to 1.18 ± 0.01 [16].

### 2.2. Preparation of GO Stock Suspensions for the Ecotoxicity Assays

Before ecotoxicity testing, the pristine GO suspension was diluted to 100 mg/L using distilled water, then it was autoclaved in 250 mL screw-cap laboratory bottles at 121 °C and 1.2 bar for 10 min. The heat-sterilized GO suspensions were stored at room temperature in the dark until use. Before all subsequent analyzes, the graphene oxide suspensions were sonicated in an ice bath using a BRANSON 450 device (400 W, 30%) for 30 min.

### 2.3. Ecotoxicity Testing

#### 2.3.1. Aliivibrio Fischeri Cultures and Test Protocol

*Aliivibrio fischeri* NRRL B-11177 was employed in the experiments. An overnight culture was obtained by inoculating 40 mL of liquid Photobacterium medium and incubating for 18 h at 24 °C in darkness with agitation at 160 rpm. The components of the Photobacterium medium were 30 g of NaCl, 6.1 g of NaH_2_PO_4_·H_2_O, 2.75 g of K_2_HPO_4_, 0.204 g of MgSO_4_·7H_2_O, 0.5 g of (NH_4_)_2_HPO_4_, 5 g of peptone, 0.5 g of yeast extract, 3 mL of glycerol per 1 L of distilled water, and pH = 7.2 [11].

The experiments were conducted in borosilicate test tubes with a test volume of 5 mL, using four replicates. A two-fold dilution series of the GO stock suspension was prepared with sterile distilled water, while distilled water served as the negative control. In the test systems, final concentrations of 0.625, 1.25, 2.5, 5, 10, 20, and 40 mg/L were set (4 mL bacterial cell suspension in Photobacterium medium + 1 mL GO sample). Each treatment had eight replicates. The assembled test systems were incubated at 22 °C in the dark and agitated at 400 rpm.

From the assembled test systems 200 µL sample was transferred into the wells of microplates in two replicates, and the luminescence intensity was measured after 3 and 9 h of exposure time using a Fluostar Optima (BMG Labtech, Ortenberg, Germany) microplate reader. Optical density was determined at 630 nm using a DIALAB ELISA EL800 (Dialab GmbH, Wiener Neudorf, Austria) microplate reader.

#### 2.3.2. Algae and Cyanobacteria Cultures and Test Protocol

The *Chlorella vulgaris*, *Chlamydomonas reinhardtii*, *Desmodesmus subspicatus* and *Synechococcus elongatus* cultures were provided by the Mosonmagyaróvár Algal Culture Collection (MACC) of the Széchenyi István University, Department of Plant Sciences. The unicellular green algae species were cultivated in Bristol liquid medium [17], while the S. elongatus cyanobacterium species was cultivated in BG medium [18] at 21.5 ± 1 °C in a thermostatic chamber under a 16:8 h light-dark cycle (illumination: Juwel Aquarium, Day-Lite, 15 W, 438 mm lamp, 560 lumens, 6500 K). The algae and cyanobacterium growth inhibition tests were conducted based on the OECD Guideline 201 [19].

#### 2.3.3. Daphnia Magna Cultures

The in-house *Daphnia magna* colony used for ecotoxicity tests was maintained in 2 L beakers at 21.5 ± 1 °C in a thermostatic chamber under a 16:8 h light-dark cycle (illumination: Juwel Aquarium, Day-Lite, 15 W, 438 mm lamp, 560 lumens, 6500 K). The colony was fed three times per week ad libitum with *Chlorella vulgaris* (2.0 mg DW/L). Boiled and cooled tap water was used for *D. magna* maintenance. The colony’s sensitivity was assessed every six months. To this purpose, potassium dichromate (K_2_Cr_2_O_7_) was used as a reference toxicant, and its response fell within the sensitivity limits (EC_50_, 24 h = 0.6–2.1 mg/L) established by OECD Guideline 202 [20].

#### 2.3.4. Daphnia Magna Reproduction Test

Ten juvenile (<24 h old) *D. magna* individuals per treatment were individually placed in 25 mL of test medium. The daphnids were exposed for 24 days to nominal GO concentrations of 0.625, 1.25, 2.5, 5, or 10 mg/L. As negative control, distilled water was applied. The semi-static reproduction tests were conducted following the OECD Guideline 211 [21]. Daphnids were fed daily with *C. vulgaris* in an age-dependent manner (50–100 μg C/test organism). The test medium was replaced every 48 h, and adult daphnids were carefully transferred to fresh medium using a special fabric spoon. Offspring were counted and removed daily. At the end of the experiment, adult heart rate, feeding activity, and body length were determined, as well as the following reproductive parameters: longevity, total number of living offspring, time to production of first eggs, time to production of first brood, and average size of broods per organism.

#### 2.3.5. Daphnia Magna Heart Rate

After 21 days of exposure, each adult individual was carefully placed on a single-cavity microscope slide in a single droplet (~30 µL) of test suspension with the help of a 10 mL volume automatic pipette with a cut pipette tip to avoid damage or disturbance of the daphnids due to the original small cross-sectional area of the tip. The heart rate of the daphnids was recorded for 10 s, but importantly, within a maximum of 30 s after placing them on the microscope slide under a NIKON SMZ800 stereomicroscope (32× magnification) [22].

#### 2.3.6. Daphnia Magna Feeding Activity

Feeding activity was determined according to the method of Kamaya et al. [23] with major modifications. After 21 h of exposure to the tested GO concentrations, the groups of daphnids were transferred to 10 mL of a 3 µL/mL concentration fluorescent microsphere suspension (Life Technologies; Waltham, Massachusetts, USA, FluoSpheres™ Carboxylate-Modified Microspheres, 0.2 µm, yellow-green fluorescent (505/515), 2% solids) diluted with the original *D. magna* growth medium. After a 20 min period in the fluorescent microsphere suspension, the daphnids were taken out with a special fabric spoon and washed thoroughly with distilled water to remove the microspheres adhered to their carapace and appendages to avoid bias in the results and to detect only the amount of microbeads that are released from the digestive tract of the daphnids due to sonication. The washed individuals were transferred to micro test tubes containing 1 mL of distilled water, then homogenized for 5 s with Sonoplus HD 4100 homogenizer (BANDELIN electronic GmbH & Co. KG, Berlin, Germany) with the following settings: frequency: 19,800 Hz, amplitude: 30%. The homogenized Daphnia-microsphere suspensions were pipetted into four parallel wells of a white 96-well flat-bottom microtiter plate, and the fluorescence intensity of the wells was measured by the FLUOstar Optima microplate reader (BMG LABTECH GmbH, Ortenberg, Germany) using the excitation wavelength of 485 nm and the emission wavelength of 520 nm.

#### 2.3.7. Daphnia Magna Body Length

After 21 h of exposure to the tested GO concentrations, the body length of each individual was measured as the distance from the eyespot to the base of the dorsal spine. Measurements were taken from digital images captured with a Nikon SMZ800 stereomicroscope at 20× magnification and analyzed using Image-Pro^®^ Plus 7.0 software.

### 2.4. Data Evaluation and Statistical Analysis

Inhibition percentages (H%) were calculated relative to the control for each ecotoxicological endpoint. Statistical analysis was performed using one-way analysis of variance (ANOVA) with STATISTICA 13^®^ software to identify significant effects (*p* < 0.05). The homogeneity of variances was examined with Cochran’s C test. Statistical significance between different treatments or dilutions was determined using the Newman–Keuls test (*p*  <  0.05). In the figures, significant effects are indicated by letters in alphabetical order, with ‘*a*’ denoting the lowest mean value. Treatments assigned the same letter indicate no significant differences between them. The Effective Concentration values (EC_20_ and EC_50_) were calculated with the OriginPro 2018 software by fitting the data to a logistic function (y = A_2_ + (A_1_−A_2_)/(1 + (x/x_0_)p)).

## 3. Results and Discussion

### 3.1. Results of the A. fischeri Bioluminescence Inhibition Test

**Figure 1:**
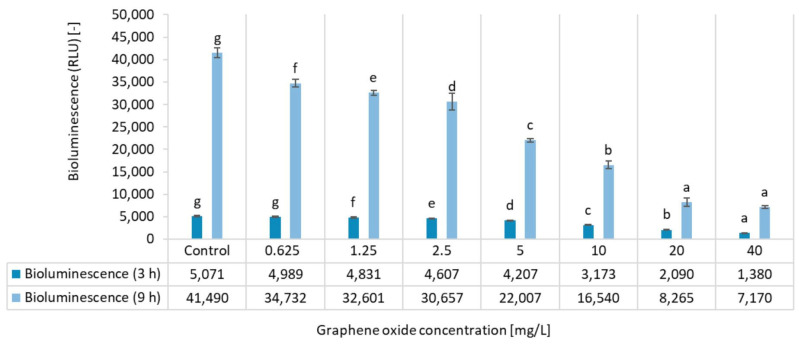
The effect of GO on the bioluminescence of *Aliivibrio fischeri* after 3 h and 9 h of exposure. Significant differences between concentrations (*p* < 0.05) distinctively for each exposure time are indicated by lowercase letters, where “*a*” represents the lowest mean value. Treatments marked with the same letter showed no significant differences.

Our results demonstrated a clear, concentration-dependent impact of GO on *A. fischeri*, with significant inhibition of bioluminescence observed at the investigated concentration range (Figure 1). Figure 1 also shows that the exposure time considerably affects the bioluminescence. Inhibition was higher after a 9 h exposure period than after a 3 h exposure period. Even the lowest tested concentration of GO (0.625 mg/L) had a significant effect compared to the control after 9 h.

The effective concentration values (EC_20_ and EC_50_), determined based on the concentration-response relationship (Table 1), also reflect both the sensitivity of the test organism and the greater inhibition of bioluminescence occurring as a result of longer exposure times. The effective concentration that resulted in 50% inhibition (EC_50_) was determined to be 14.92 mg/L following an exposure time of 3 h. However, this concentration was found to be 6.05 mg/L when the contact time was extended to 9 h.

The lowest observed effect concentration (LOEC), which differed significantly from the control group, was also lower after 9 h of exposure (Table 1).

As far as we are aware, there are no examples in the literature of chronic effect assessments of graphene-based nanomaterials using the *A. fischeri* bioluminescence inhibition test. Consequently, this study is novel in this respect, as it can provide new data for the environmental risk assessment of graphene oxides. In their complex impact assessment, Németh and colleagues [11] determined an EC_20_ value of 5.06 mg/L in the *A. fischeri* bioluminescence inhibition acute toxicity tests at 30 min contact time conducted with graphene oxide PM 995. The acute toxicity assay of graphene-derived materials on *A. fischeri* using the Microtox test also exhibited lower sensitivity and toxicity, with EC_20_ values ranging from 1.42 to >5.00 mg/L at short exposure times (5–30 min) [8]. The toxicity mechanisms impacting *A. fischeri*, as reflected by the inhibition of bioluminescence, may involve membrane damage, the generation of reactive oxygen species (ROS) leading to cell death, and/or electrostatic interactions [8,11,24].

The higher toxicity and lower effective concentration values (e.g., LOEC = 0.625 mg/L) obtained in our chronic test clearly illustrate the greater sensitivity of chronic endpoints and the necessity to evaluate long-term effects.

### 3.2. Results of the Algae and Cyanobacteria Growth Inhibition Tests

The calculated inhibition percentage values clearly demonstrated a concentration-dependent effect across all algal and cyanobacterial species tested (Table 1). However, the magnitude of inhibition varied substantially between species, indicating species-specific sensitivity to GO. For *C. reinhardtii*, inhibition increased progressively from 40% at 3.125 mg/L to 79% at 50 mg/L. Therefore, this species exhibited a moderate sensitivity level, with inhibition values rising steadily but not reaching the highest levels compared to the other organisms. *C. vulgaris* was the most sensitive species in the test. Inhibition was already 72% at the lowest concentration of 3.125 mg/L; values exceeded 85% from 25 mg/L onwards. This pattern indicates that *C. vulgaris* responds strongly even at low exposure levels. In contrast, *D. subspicatus* showed the lowest sensitivity. Inhibition ranged from 17% at 3.125 mg/L to 50% at 50 mg/L. The values plateaued between 12.5 and 25 mg/L, both showing 42% inhibition, suggesting a tolerance threshold or potential adaptive mechanisms that limit further inhibition at intermediate concentrations.

*S. elongatus* displayed a different response pattern compared to the green algae. Inhibition was relatively low at the two lowest concentrations, ranging from 9% to 31%, but increased sharply at higher concentrations, reaching 86% at 50 mg/L. This indicates that *S. elongatus* is relatively resistant to low levels of GO exposure even after 168 h of exposure, but shows strong sensitivity once a certain concentration threshold is exceeded.

**Figure 2:**
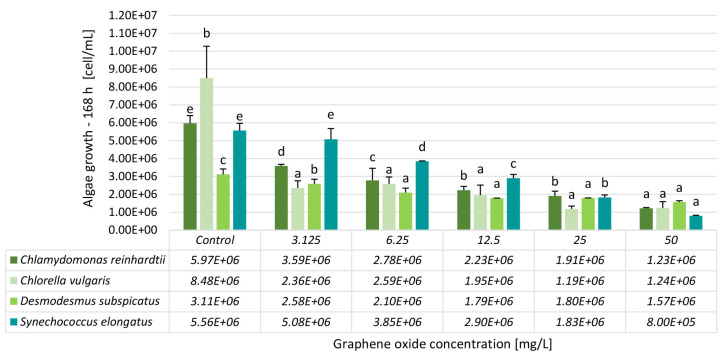
The effect of GO on the growth of unicellular green algae (C*. reinhardtii, C. vulgaris, D. subspicatus*) and a cyanobacterium species (*S. elongatus*) after 168 h of exposure. Significant differences between concentrations (*p* < 0.05) distinctively for each test organism species are indicated by lowercase letters, where “*a*” represents the lowest mean value. Treatments marked with the same letter showed no significant differences.

When comparing the four species, *C. vulgaris* was the most sensitive organism at low concentrations, followed by *C. reinhardtii*, *D. subspicatus*, and *S. elongatus* (Figure 2). At the highest concentration tested, *S. elongatus* and *C. vulgaris* showed the strongest inhibition (86% and 85%, respectively), followed by *C. reinhardtii* (79%) and *D. subspicatus* (50%).

Differences in algal and cyanobacterial sensitivity to GO were explained by differences in cell size, morphology, cell wall composition, and the presence of flagella that directly alter GO-induced shading, mechanical damage, or surface envelopment. For example, no apparent mechanical injury was detected on *C. vulgaris* or *C. reinhardtii* after GO exposure for 96 h, likely due to their protective cell wall composition [25]. Malina et al. [26] also described a “nano-blade” effect in the case of green algae and *S. elongatus*, responsible for membrane integrity damage. In their study, the cyanobacterium *S. elongatus* showed greater sensitivity to GO than green algae, consistent with the well-documented antibacterial properties of GO. Toxic effects were mainly attributed to shading, membrane damage, and depletion of nutrients; however, it has been revealed by a detailed mechanistic analysis that GO affected *Raphidocelis subcapitata* through an additional, previously unrecognized pathway. Notably, lightly oxidized GO caused significantly more substantial damage to cell membrane integrity than more heavily oxidized variants [26].

In the current scientific literature, EC_50_ values for GO toxicity in algal growth inhibition tests are predominantly reported for 72 h or 96 h exposure conditions. EC_50_ values were determined as follows: *R. subcapitata* (96 h) ~20 mg/L [27], *Scenedesmus obliquus* (72 h) 20.6 mg/L [28], *C. vulgaris* (96 h) 37.52 mg/L [29], *Chlorella pyrenoidosa* (96 h) 37.3 mg/L [12].

In contrast, our study applied a prolonged exposure period of 168 h, which revealed substantially different sensitivity patterns. We observed EC_50_ values of 2.52 ± 0.28 mg/L for *C. vulgaris*, 50 ± 0.8 mg/L for *D. subspicatus*, 5.82 ± 0.36 mg/L for *C. reinhardtii*, and 14.79 ± 0.41 mg/L for *S. elongatus* (Table 2). These findings suggest that extended exposure increases the discriminatory power of the test, highlighting species-specific responses that may be underestimated under shorter, standard test durations.

### 3.3. Results of the D. magna Heart Rate Test

**Figure 3:**
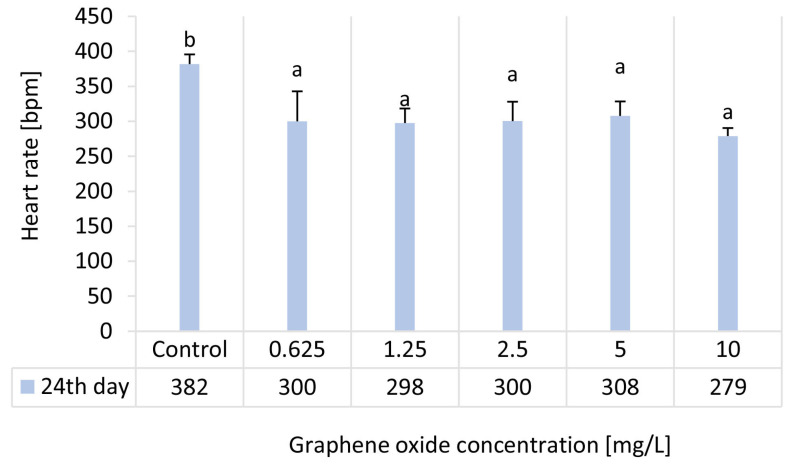
The effect of GO on the heart rate of adult *D. magna* individuals in beats per minute (bpm) units after 24 days of exposure. Significant differences between treatments (*p* < 0.05) are indicated by lowercase letters, where “*a*” represents the lowest mean value. Treatments marked with the same letter showed no significant differences.

At the end of the chronic *D. magna* test, the heart rate of adult daphnids was measured. All tested GO concentrations (0.625–10 mg/L) caused a statistically significant inhibitory effect compared to the control. However, within the tested concentration range, statistical analysis revealed no significant differences among treatments. (Figure 3). The highest heart rate inhibition was observed at the highest GO concentration tested (H% = 26.9), whereas in the 0.625–5 mg/L range, inhibition values varied between 19.4% and 22.0% (Table 3).

Although *Daphnia* heart rate has been widely employed as a sensitive sublethal physiological endpoint in ecotoxicological research [30,31,32,33,34], its application in the assessment of nanomaterial toxicity remains relatively scarce [31,35,36,37], particularly in the case of carbon-based nanomaterials (CNMs) [38] such as graphene oxide [22]. To the best of our knowledge, the only study assessing the effects of GO on the heart rate of *D. magna* is our previous work [22], in which significant inhibition was observed at 25 and 50 mg/L after 48 h exposure (H% = 25.5 and 43.4, respectively). However, in the present study, GO was tested over a prolonged exposure period of 24 days, at the applied lower concentrations (0.625–10 mg/L), statistically significant heart rate inhibition was found, ranging between 19.4% and 26.9%. As one of the main underlying mechanisms of GO toxicity in *D. magna* was attributed to oxidative stress, we hypothesize that the described decrease in heart rate may be directly related to ROS production induced by exposure to GO, as this phenomenon has been described previously [39,40].

### 3.4. Results of the D. magna Feeding Activity Test

**Figure 4:**
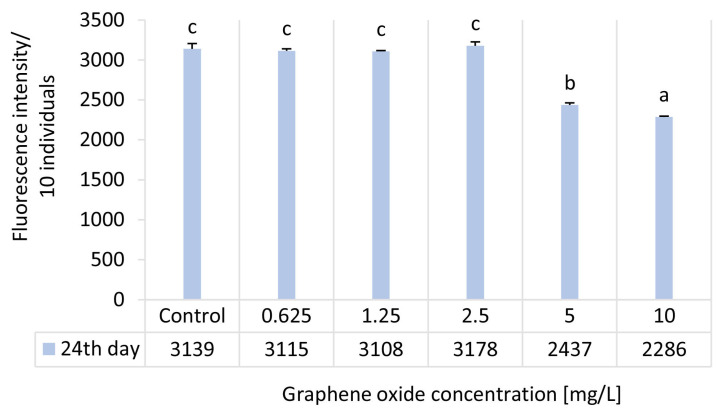
The effect of GO on the feeding activity of adult *D. magna* individuals after 24 days of exposure. Significant differences between treatments (*p* < 0.05) are indicated by lowercase letters, where “*a*” represents the lowest mean value. Treatments marked with the same letter showed no significant differences.

In the case of the feeding activity test, significant inhibition compared to the control was observed only at the two highest GO concentrations (5 and 10 mg/L), with inhibition percentage values of 22.4% and 27.2%, respectively (Table 3). No statistically significant differences were detected at lower concentrations within the tested range (Figure 4). However, some previous studies reported findings on the effect of GO on *D. magna* feeding and depuration; a direct comparison cannot be made due to differences in test duration, applied GO concentrations, feeding strategies, as well as the product-specific characteristics of the tested GO samples [41,42,43,44]. Acute ecotoxicity results of our previous study on the effect of the same GO product showed that exposure of *Daphnia magna* for 48 h within the 3.125–50 mg/L concentration range caused a significant reduction in feeding activity. At the highest concentration (50 mg/L), feeding activity decreased by approximately 91%. Within the 6.25–25 mg/L range, inhibition ranged from 30.7% to 36.6%. The observed reduction in feeding activity of *Daphnia magna* upon GO exposure can be attributed to oxidative stress localized in the digestive tract [22,45], as well as impairments in swimming velocity caused by nanoparticle adhesion to the antennae [46] and altered thoracic appendage movement disrupting the filter-feeding mechanism [47]. The observed reduction in feeding activity of *Daphnia magna* upon GO exposure can be attributed to oxidative stress localized in the digestive tract [22,45], as well as impairments in swimming velocity caused by nanoparticle adhesion to the antennae [46] and altered thoracic appendage movement disrupting the filter-feeding mechanism [47].

### 3.5. Results of Body Length Determination of D. magna

**Figure 5:**
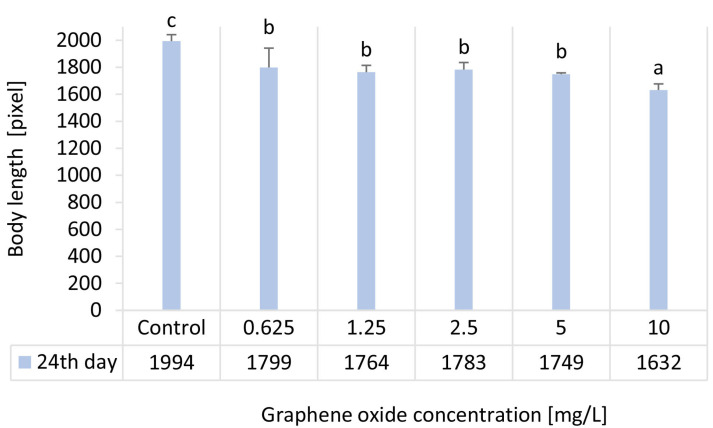
The effect of GO on the body length of adult *D. magna* individuals after 24 days of exposure. Significant differences (*p* < 0.05) between treatments (*p* < 0.05) are indicated by lowercase letters, where “*a*” represents the lowest mean value. Treatments marked with the same letter showed no significant differences.

Results of the adult *D. magna* body length measurements showed that all tested GO concentrations (0.625–10 mg/L) caused a statistically significant inhibitory effect compared with the control. Exposure to 10 mg/L GO resulted in an 18.2% reduction (Table 3) in adult body length compared with the control, whereas in the 0.625–5 mg/L range, the average body length decreased by 10.6–12.9% (Figure 5). The observed reduction in body length is closely associated with altered physiological functions, indicating a strong relationship between reduced growth and decreases in *D. magna* feeding activity and heart rate in.

On the contrary, in a 21-day chronic toxicity test, exposure to pegylated GO at concentrations ranging from 2 to 400 µg/L did not result in significant differences in *Daphnia magna* body length compared to the control group [44]. Liu et al. [43] investigated the chronic toxicity of pristine GO and its functionalized derivatives (GO-polyethylene glycol, GO-carboxyl, andGO-imidazole) at 1 mg/L concentration on *D. magna* over two generations and found that while F0 individuals showed no adverse effects on body length or reproduction, significant inhibition in growth and reproduction appeared in the F1 generation, indicating that potential long-term effects may not be evident during initial exposure.

### 3.6. Results of the D. magna Reproduction Test

**Figure 6:**
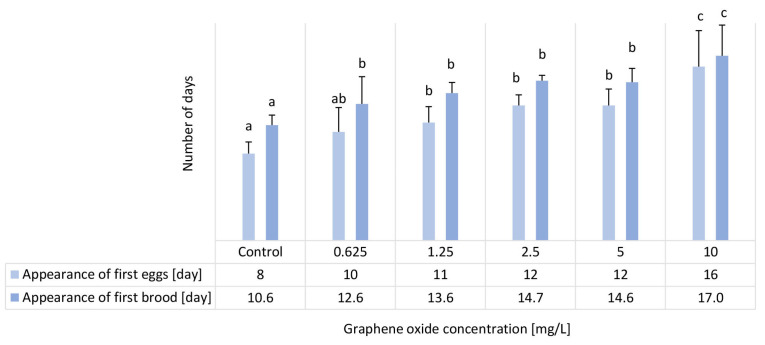
The effect of GO exposure on the appearance of first eggs and brood in the 24-day semi-static *D. magna* reproduction test. Significant differences between treatments (*p* < 0.05) are indicated by lowercase letters, where “*a*” represents the lowest mean value. Treatments marked with the same letter showed no significant differences.

**Figure 7:**
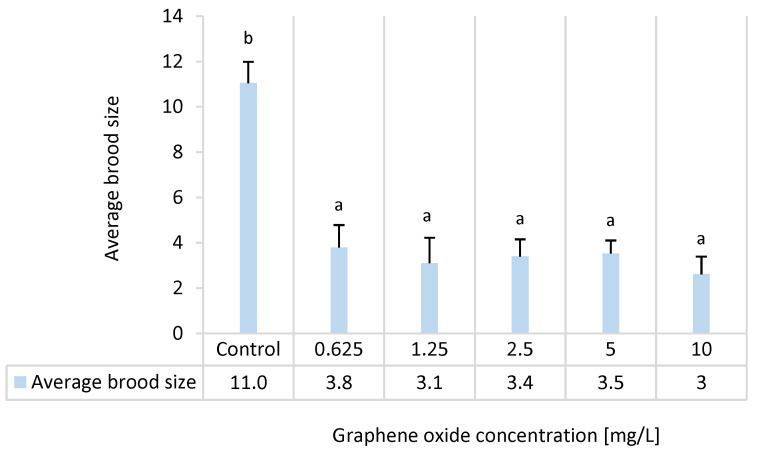
The effect of GO exposure on average brood size in the 24 d semi-static *D. magna* reproduction test. Significant differences between treatments (*p* < 0.05) are indicated by lowercase letters, where “*a*” represents the lowest mean value. Treatments marked with the same letter showed no significant differences.

Due to GO exposure, a significant delay was experienced in the appearance of first eggs in the 1.25–10 mg/L concentration range. In contrast, a significant delay was experienced in the appearance of the first brood in all tested concentrations in a concentration-dependent manner (Figure 6). Average brood size was dramatically decreased (3–3.8 offspring/brood) due to GO exposure in all tested concentrations compared to the control value of 11 offspring/brood. This parameter did not show a concentration-dependent manner (Figure 7).

Liu et al. [43] conducted a two-generation chronic exposure study of *D. magna* to pristine GO and three functionalized derivatives (carboxyl-, imidazole-, and polyethylene glycol-modified GO) at a concentration of 1 mg/L over 21 days. They reported that the appearance of the first brood was delayed by approximately 2 days under pristine GO exposure, while functionalization reduced this delaying effect. The extent of mitigation followed the order: carboxyl > imidazole > polyethylene glycol (PEG-GO), with the PEG-GO treatment showing no statistically significant delay compared to control. This suggests that surface functionalization alters the bioavailability and interaction of GO with daphnids, thereby modifying its chronic reproductive toxicity. Zhang et al. [28] also reported a slight, statistically significant delay in the appearance of the first brood at 0.01–1 mg/L GO concentrations. Interestingly, according to their results, no first brood was observed during the 21-day experimental period at a GO concentration of 10 mg/L.

In comparison, pristine GO induced stronger effects on reproductive timing in our study. At 1.25 mg/L, the first brood was delayed by approximately 3 days, which is more pronounced than the ~2-day delay observed by Liu et al. [43] at a slightly lower concentration. Moreover, the delaying effect in our case was clearly concentration-dependent: 2.5 mg/L caused a 4-day delay, while the highest tested concentration (10 mg/L) postponed the appearance of the first brood by about 6.5 days. These findings indicate that the magnitude of delay may vary depending on experimental conditions (e.g., exposure setup, particle properties, medium composition), and functionalization can substantially reduce or abolish this effect. Our results therefore reinforce the conclusion that surface chemistry strongly governs the chronic toxicity of GO, while also demonstrating that under certain conditions pristine GO can cause more severe reproductive impairment than previously reported.

**Figure 8:**
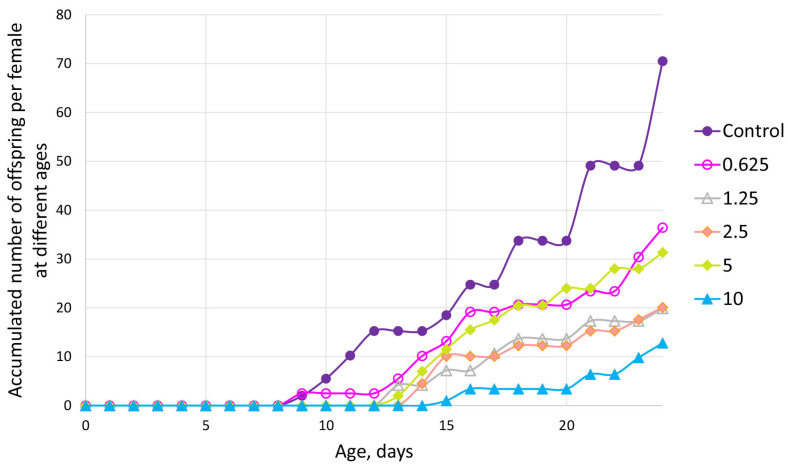
Cumulative mean offspring per test-organism exposed to GO at different time intervals during a 24 d semi-static *D. magna* reproduction test.

The cumulative mean number of offspring per female *D. magna* was strongly affected by exposure to GO over the 24-day test period. In the control group, reproduction began around day 9 and increased steadily, reaching approximately 70 offspring per female by the end of the test. At the lowest GO concentration (0.625 mg/L), reproduction was reduced compared to the control but reached around 40 offspring per female (Figure 8). Reproduction was further suppressed at intermediate concentrations (1.25 and 2.5 mg/L), with the cumulative number of offspring plateauing at around 20. Interestingly, reproduction at 5 mg/L resulted in a slightly higher output (~30 offspring) than at 2.5 mg/L, suggesting some variability or a potential non-linear concentration–response relationship. Reproduction was strongly inhibited at the highest tested concentration (10 mg/L), with females producing fewer than 15 offspring on average. Overall, the results demonstrate that GO exposure negatively impacts *D. magna* reproduction in a concentration-dependent manner, with the strongest effects observed at 10 mg/L. The observed deviation from strict dose dependency between 2.5 and 5 mg/L may indicate biological variability or complex interactions between GO and the test organisms.

Liu et al. [43] did not find statistically significant differences in the cumulative number of offspring compared to control when exposed to 1 mg/L GO for 21 d. At the same time, Zhao et al. [12] reported a significant concentration-dependent decrease in the cumulative number of offspring, similar to our findings. Souza et al. [48] investigated the chronic effects of graphene oxide on the cladoceran *Ceriodaphnia dubia*, a close relative of *D. magna*. After seven days of exposure, they reported a significant reduction in the cumulative number of offspring at the two highest tested concentrations. Specifically, neonate production was reduced by 12.8% at 0.4 mg/L and 44.2% at 0.8 mg/L. These results clearly demonstrate that GO can impair reproductive output in cladocerans, with substantial decreases in neonate production occurring even at sub-milligram per liter concentrations.

The potential environmental risk can be determined by applying the EU Technical Guidance Document (TGD) approach [49] using the Risk Characterisation Ratio (RCR). The RCR is defined as the ratio of the Predicted Environmental Concentration (PEC) to the Predicted No Effect Concentration (PNEC). The RCR value (RCR = PEC/PNEC) indicates the potential adverse impact of a substance on the ecosystem.

The PNEC is calculated using ecotoxicological data, with the lowest effective concentration value divided by assessment factors ranging from 10 to 1000 depending on the reliability and availability of the ecotoxicity test results [11,49]. Despite examining chronic effects, we applied an extrapolation factor of 1000 in accordance with the objective of adopting a conservative approach to ensure environmental safety, considering the available test data. Using the lowest effective concentration value (LOEC = 0.625 mg/L in the *A. fischeri* bioluminescence test) and applying a safety assessment factor of 1000, the PNEC value was determined to be 625 ng/L.

Regarding environmental concentrations, we used literature data to determine the PEC value. Hong and Nowack [50] predicted environmental concentrations of 0.33 ng/L for graphene oxide derivatives in European freshwaters by 2030.

According to our study, the RCR value for nGO PM 995 was calculated to be 5.3 × 10^−4^, which is significantly lower than 1 and indicates a low risk to freshwater aquatic systems.

## 4. Conclusions

In accordance with the objectives of our innovative research, a comprehensive methodology based on chronic effect assessment was applied to evaluate the environmental impact and potential risks of a graphene oxide product. Our approach encompasses different trophic levels and innovative endpoints not previously covered in the literature, providing new data to support environmental risk assessment of graphene-based nanomaterials. Based on the results of our comprehensive assessment and on the EU TGD guidelines, we conclude that the tested graphene oxide product does not pose an environmental risk based on our results in the applied test systems, even when applying a conservative environmental risk assessment approach, i.e., the worst-case scenario.

## Figures and Tables

**Table 1:** Inhibition percentage values calculated for the bioluminescence inhibition test results after 3 h and 9 h of exposure.

Inhibition [%]		
Concentration [mg/L]	3 h of Exposure	9 h of Exposure
0.625	2 ± 2	16 ± 2
1.25	5 ± 2	21 ± 1
2.5	9 ± 2	26 ± 4
5	17 ± 1	47 ± 1
10	37 ± 2	60 ± 2
20	59 ± 1	80 ± 2
40	73 ± 2	83 ± 1
EC_20_	5.36 ± 0.27	1.26 ± 0.06
EC_50_	14.92 ± 0.75	6.05 ± 0.30
LOEC	1.25	0.625

**Table 2:** Inhibition percentage values calculated for the algae and cyanobacteria growth inhibition test results after 168 h of exposure.

Inhibition [%]—168 h				
Conc. [mg/L]	*Chlamydomonas reinhardtii*	*Chlorella vulgaris*	*Desmodesmus subspicatus*	*Synechococcus elongatus*
3.125	40 ± 1	72 ± 5	17 ± 2	9 ± 1
6.25	53 ± 13	70 ± 5	33 ± 4	31 ± 0
12.5	63 ± 6	77 ± 7	42 ± 0	48 ± 4
25	68 ± 9	86 ± 2	42 ± 0	67 ± 5
50	79 ± 2	85 ± 4	50 ± 2	86 ± 4
EC_20_	1.38 ± 0.09	0.7 ± 0.25	3.65 ± 0.06	5.34 ± 0.15
EC_50_	5.82 ± 0.36	2.52 ± 0.28	50 ± 0.8	14.79 ± 0.41

**Table 3:** Physiological test endpoint results given in inhibition percentage values in the 24-day semi-static *D. magna* reproduction test.

Ecotoxicity Test Endpoint		Inhibition [%]—24 d			
		Graphene Oxide Concentration [mg/L]			
	0.625	1.25	2.5	5	10
Heart rate	21.4 ± 11.2	22 ± 5.4	21.3 ± 7.3	19.4 ± 5.4	26.9 ± 3.0
Feeding activity	0.8 ± 0.8	1 ± 0.3	−1.2 ± 1.6	22.4 ± 0.9	27.2 ± 0.3
Body length	12.9 ± 4.2	12.1 ± 2.8	10.6 ± 2.6	12.3 ± 0.5	18.2 ± 4.3

EC_20_ values calculated for the physiological test endpoints were 0.73 mg/L for heart rate, 5.06 mg/L for feeding activity and >10 mg/L for body length. EC_50_ values could not be determined, as inhibition did not reach 50% for any of the applied ecotoxicity endpoints.

## Data Availability

The data presented in this study are available on request from the corresponding author.

## Abbreviations

The following abbreviations are used in this manuscript:

EC	Effective Concentration
GO	*Graphene Oxide*
LOEC	Lowest Observed Effect Concentration
NOEC	No Observed Effect Concentration
PEC	Predicted Environmental Concentration
PNEC	Predicted No Effect Concentration
RCR	Risk Characterisation Ratio
